# Immunomodulatory Role of Nutrients: How Can Pulmonary Dysfunctions Improve?

**DOI:** 10.3389/fnut.2021.674258

**Published:** 2021-09-07

**Authors:** Sarah Cristina Gozzi-Silva, Franciane Mouradian Emidio Teixeira, Alberto José da Silva Duarte, Maria Notomi Sato, Luana de Mendonça Oliveira

**Affiliations:** ^1^Laboratório de Dermatologia e Imunodeficiências (LIM-56), Departamento de Dermatologia, Instituto de Medicina Tropical, Faculdade de Medicina da Universidade de São Paulo - FMUSP, São Paulo, Brazil; ^2^Departamento de Imunologia, Instituto de Ciências Biomédicas, Universidade de São Paulo, São Paulo, Brazil; ^3^Departamento de Patologia, Faculdade de Medicina da Universidade de São Paulo - FMUSP, São Paulo, Brazil

**Keywords:** nutrients, lung health, pulmonary chronic diseases, asthma, COPD, SARS-CoV-2 infection, COVID-19

## Abstract

Nutrition is an important tool that can be used to modulate the immune response during infectious diseases. In addition, through diet, important substrates are acquired for the biosynthesis of regulatory molecules in the immune response, influencing the progression and treatment of chronic lung diseases, such as asthma and chronic obstructive pulmonary disease (COPD). In this way, nutrition can promote lung health status. A range of nutrients, such as vitamins (A, C, D, and E), minerals (zinc, selenium, iron, and magnesium), flavonoids and fatty acids, play important roles in reducing the risk of pulmonary chronic diseases and viral infections. Through their antioxidant and anti-inflammatory effects, nutrients are associated with better lung function and a lower risk of complications since they can decrease the harmful effects from the immune system during the inflammatory response. In addition, bioactive compounds can even contribute to epigenetic changes, including histone deacetylase (HDAC) modifications that inhibit the transcription of proinflammatory cytokines, which can contribute to the maintenance of homeostasis in the context of infections and chronic inflammatory diseases. These nutrients also play an important role in activating immune responses against pathogens, which can help the immune system during infections. Here, we provide an updated overview of the roles played by dietary factors and how they can affect respiratory health. Therefore, we will show the anti-inflammatory role of flavonoids, fatty acids, vitamins and microbiota, important for the control of chronic inflammatory diseases and allergies, in addition to the antiviral role of vitamins, flavonoids, and minerals during pulmonary viral infections, addressing the mechanisms involved in each function. These mechanisms are interesting in the discussion of perspectives associated with severe acute respiratory syndrome coronavirus 2 (SARS-CoV-2) infection and its pulmonary complications since patients with severe disease have vitamins deficiency, especially vitamin D. In addition, researches with the use of flavonoids have been shown to decrease viral replication *in vitro*. This way, a full understanding of dietary influences can improve the lung health of patients.

## Introduction

The lungs are fundamental organs of the respiratory system, whose main function involves extracting oxygen from the environment and making it available for aerobic respiration at the cellular level. Oxygen is used for the synthesis of ATP (adenosine triphosphate) and carbon dioxide is eliminated with other metabolic by-products (1, 2). However, in addition to their primarily respiratory functions, they are also important in other non-respiratory processes. A group of lungs cells, pulmonary neuroendocrine cells (PNECs), is responsible for the secretion of a variety of amines and peptides playing an important role in cell growth and differentiation (3). It is also an essential organ for the degradation and inactivation of chemical mediators, (4) in addition to participating in the optimization of cardiac output (3). There is also a cross-talk between intestine-lung that influences the maintenance of pulmonary mucosa homeostasis, as well as the response against pathogens and the development of inflammatory diseases (5).

The lungs are chronically exposed to various pathogenic or non-pathogenic environmental antigens. Therefore, maintaining a network of resident cells that continuously monitor the external environment and promote tolerance to innocuous particles is essential for pulmonary homeostasis. On the other hand, the deficiency in the immune response counts pathogens or intense inflammatory responses as a result of failures of mechanisms of tolerance, can generate damage to the tissue and lung function, contributing to the development of chronic inflammatory diseases for exemple chronic obstructive pulmonary disease (COPD) and asthma, and infections (6).

According to data from the World Health Organization (WHO), COPD is the third leading cause of death worldwide, causing 3.23 million deaths in 2019, with more than 80% of these deaths occurred in low- and middle-income countries (7, 8). Asthma is one of the main non-communicable diseases, affecting both children and adults. In 2019, about 262 million were affected by asthma and 461,000 people died (9). In addition to inflammatory lung diseases, lower respiratory infections continue to be the deadliest communicable disease in the world, ranked as the 4th leading cause of death. In 2019, 2.6 million individuals died (7). Citing examples of respiratory viral infections, it is estimated that annual influenza epidemics result in about 3 to 5 million cases of serious illness and about 290,000–650,000 respiratory deaths (10). In the current scenario, severe acute respiratory syndrome coronavirus 2 (SARS-CoV-2) infection stands out, which mainly affects the lungs, in addition to other organs, and has already been responsible for more than 4.2 million deaths around the world (11). These data reinforce the importance of studies aimed at pulmonary health.

In this context, diet and nutrition are modifiable contributors to the development and progression of chronic diseases and lung infections. There is considerable evidence that indicates the importance of food intake in obstructive pulmonary diseases, such as asthma and COPD both in early life and in the development of the disease, as well as the importance of nutrition in the response against pulmonary infections (12). Macronutrients, micronutrients and bioactive components can influence homeostasis and protection of exacerbated inflammatory responses in lung tissue (13).

In this review, we discuss the interaction between nutrients and inflammatory (asthma and COPD) and infectious (viral infections) lung conditions. General physiological and immunological characteristics of the lungs were also reviewed, as well as perspectives on the possible immunomodulatory potential of some nutrients in SARS-CoV-2 infection.

## Lung General Characteristics and Pulmonary Immunology

The lungs are a pair of primary respiratory organs present in the chest cavity next to the mediastinum. They are covered by a thin double-layer serous membrane, the pleura (14). The conduction portion of the lungs begins at the trachea and extends to the terminal bronchioles, providing a pathway for movement and conditioning of the air entering the lungs (15).

The cells of the respiratory epithelium collaborate to heat, hydrate and remove incoming particles. Most of the respiratory epithelium is the ciliated pseudostratified columnar epithelium, which controls the actions of the mucociliary escalator, a primary lung defense mechanism that removes debris (16). Goblet cells are filled with mucin granules on their apical surface, and their primary function is to secrete mucin and create a protective layer of mucus (17). Basal cells, on the other hand, connect to the basal membrane and provide a fixation layer for hair and goblet cells, in addition to interacting with lymphocytes and dendritic cells (DCs) (18).

The structural and functional unit of the respiratory system consists of the alveoli, which is the main location where gas exchange for the pulmonary vasculature occurs. In an adult individual, there are ~300 million alveoli (19, 20).

The alveolar membrane is the largest surface area of the body in contact with the external environment and is continuously exposed to a wide variety of microbes and organic and inorganic particles. This constant exposure requires immunological mechanisms that tolerate innocuous particles that are inhaled and that immediately defend the host from microbial products or pathogens that can enter the lungs (21).

The complex interaction between airway epithelial cells and immune cells, together with chemokines and soluble proteins, shapes the outcome of host-pathogen interaction within the airway microenvironment (22). The airway epithelium restricts the growth of microorganisms because the cilia present in the epithelium move fluid, mucus, and trapped particles out of the lungs. Airway fluids also contain lysozyme, lactoferrin, and antimicrobial defensins that restrict microbial growth (21). Lung epithelial cells also express pattern recognition receptors (PRRs), such as Toll-like receptors (TLRs), that recognize microbial-associated molecular patterns (MAMPs); in addition to secreting a variety of antimicrobial products, such as defensins, complement proteins and collectins, assisting in the regulation and recruitment of immune cells (23).

Particles of 1 μm in size or smaller, which correspond to the size of bacterial and viral particles, are transported to the alveolar surface, where they interact with soluble components (for e.g., IgG, complement and surfactant) that, by means of opsonization, help phagocytosis by alveolar macrophages (24, 25).

In addition, the variety of surface receptors of alveolar macrophages allows them to sense the environment and to signal to lung stromal cells, with the aim of maintaining homeostasis or allowing the perception of changes in the inhaled environment. These signals can be activators (TLR 2, 4, 6; IL-1R, IFNγR, and TNFR), generally induced during conditions of poor nutrition, infections, use of antibiotics, pollution, smoking, or limitation of the microbiota; or suppressors (CD200R, SIRPα, mannose receptor, TREM2, IL-10R, and TGFβR) that are related to homeostasis and induced during conditions of balanced diet, minimal infections, limited antibiotic usage, or diverse microbiota (23).

### Immune Cells in the Lungs

During homeostatic conditions in lung parenchyma, the immune cells are present in the following proportion: alveolar macrophages consist of ~95% pulmonary leukocytes, with 1–4% lymphocytes and ~1% neutrophils. In this context, alveolar macrophages are highly phagocytic and ingest a large number of inhaled inert particles that reach alveolar spaces, without triggering inflammatory responses, maintaining homeostasis (26). These cells are also capable to phagocytize apoptotic cells, preventing dead cells from releasing pro-inflammatory and toxic contents to the environment, while triggering the release of anti-inflammatory factors as a transforming growth factor β1 (TGF-β1) and prostaglandin E 2 (PGE 2) (27). In addition, the alveolar macrophages can also phagocytize pathogens initiating the immune response. Therefore, alveolar macrophages are phagocytic cell sentinels of the innate immune system in the lungs, providing a first line of defense and maintaining homeostasis through interaction with pulmonary epithelial cells (26).

The mucosa of the pulmonary conducting pathways has networks of DCs, especially myeloid DCs, which assist in immune surveillance and are capable of capturing antigens both within the intact epithelium and in the lumen of the airways (28).

Intraepithelial T cells (especially CD8) and T cells of the pulmonary lamina propria (especially CD4) are found in relatively high numbers in the mucosa and have an effector and/or memory phenotype, according to the expression of CD45RO, present in memory T cells (6). The lamina propria also contains some scattered B cells, which in addition to producing antibodies, can also contribute to the presentation of local antigens (6, 29).

Another important cell in pulmonary immunology is regulatory T (Tregs) cells. Tregs cells develop tolerogenic immune responses to innocuous antigens that find mucous surfaces. They express the forkhead box transcription factor P3 (FOXP3), which allows the regulation of the immune response through anti-inflammatory cytokines, such as interleukin (IL)-10 or TGF-β. The maintenance of immunological tolerance in the lungs by Tregs cells is achieved by modulating of CD4^+^ T cells subsets–T helper (Th) 1, 2, or 17 cells (30, 31). It has been shown that the inadequate response of Treg cells can lead to greater susceptibility and spread of infections. The proposed mechanism includes damage to lung tissue secondary to exacerbated T-cell activity, so Tregs cells also play an important role in lung defense against pathogens (31).

It is described that individuals with COPD who presented a rapid decline in lung function, had lower frequencies of Tregs cells in the bronchoalveolar lavage compared to individuals with COPD with a non-rapid decline, suggesting that the inability to suppress the inflammatory response may lead to a rapid decline in lung function (32). It was also reported in patients with pulmonary emphysema that the peripheral capacity of these cells was normal both in patients with emphysema and in control subjects, however, secretion of IL-10 by Tregs cells from the entire lung of patients with emphysema was impaired, suggesting that the inflammatory medium affects the activation and function of Tregs cells in local tissues (33).

Another group of lung cells, important for supporting homeostasis and maintaining tissue integrity, consists of innate lymphoid cells (ILCs). ILCs show transcriptional and functional parallels with subsets of helper T cells (Th1, Th2, Th17), with the difference that ILCs do not have specific antigen receptors clonally distributed, responding to danger signals from the mucous epithelium (34).

ILC2s play a key role in maintaining the epithelial barrier of the respiratory tract, through the production of cytokines including IL-4, IL-5, IL-9, IL-13, and the epidermal growth factor-like molecule amphiregulin (34). It was demonstrated in the lung that the depletion of ILC2 negatively affected the integrity of the epithelial barrier of the airways after infection by the influenza virus. This is because the depletion of ILC2 caused a failure to generate hyperplastic epithelial cells, leading to the deterioration of the epithelial lining (35). In the lung, IL-22 is produced especially by ILC3 and has also been shown to be involved in maintaining epithelial barrier function, mucus production, and tissue repair. Thus, ILCs contribute to barrier surveillance and epithelial protection and repair through coordinated interactions with other cells in the lung (34). On the other hand, ILCs can contribute to lung diseases by accumulating and/or altering their subsets. In patients with COPD, the signatures of IL-12 and the accumulation of ILC1s are elevated. IL-12 induces the conversion of ILC2s into interferon (IFN)-γ-producing ILC1s, thus contributing to the type 1 inflammatory lesion associated with COPD (36).

In addition to effector cells, the airway mucosa also has bronchial-associated lymphoid tissue (BALT), which comprises aggregates of lymphoid cells underlying Peyer's plaques. The presence of BALT is common in young children; however, its importance in adult humans has been questioned. It has been suggested that BALT may play a significant role in local immune homeostasis within the respiratory tract early in life, when important elements within central lymphoid structures are not fully mature (6, 37). Part of BALT role is related to the humoral immune response of the mucosa and more specifically to the production of immunoglobulin (Ig) A (IgA) (38).

IgA is the antibody isotype most present in the mucosal immune system and consists of its main defense mechanism. Provide the first line of defense in these locations against external agents without inducing a potentially harmful inflammatory response (39). Most of the IgA in the blood (about 90%) is monomeric (mIgA), while in pulmonary secretions, about half of the IgA is dimeric (dIgA), and most of it is in the form of secretory IgA (sIgA) (40).

In the infection context, sIgA helps protect the mucosal epithelium barrier through two main protective functions (41). The first, called immune exclusion, acts on the stromal epithelial portion, where IgA can form complexes with antigens. These immune complexes can be captured by phagocytic cells, absorbed into the vascular system or transported through the epithelium to the lumen. This immune elimination feature of IgA allows the maintenance of mucous tissues and protects against excess antigens that can cause infections (38). The second function is based on intracellular neutralization (41). In this case, IgA is able to prevent the assembly of the virus and neutralizes viral replication. Thus, it can interfere with the ability of antigens to adhere and penetrate the mucosa (42).

SIgA deficiency was demonstrated on the bronchial mucosa surface of ex-smokers with COPD. The deficiency was associated with latent or persistent herpesvirus infection, thickening of the submucosa and fibrotic remodeling of the airway walls (43). Associated epithelial damage also supports an inefficient first line of defense with decreased mucociliary clearance and IgA secretion (38). On the other hand, in asthma it is believed that the activation of granulocytes represents a driving force, since Fc alpha receptor (FcαR) is widely distributed in granulocytes. Thus, IgA can influence the fate of inflammatory diseases, inducing eosinophil degranulation and leading to the destruction and/or injury of the respiratory epithelium (38, 44).

**Figure 1:**
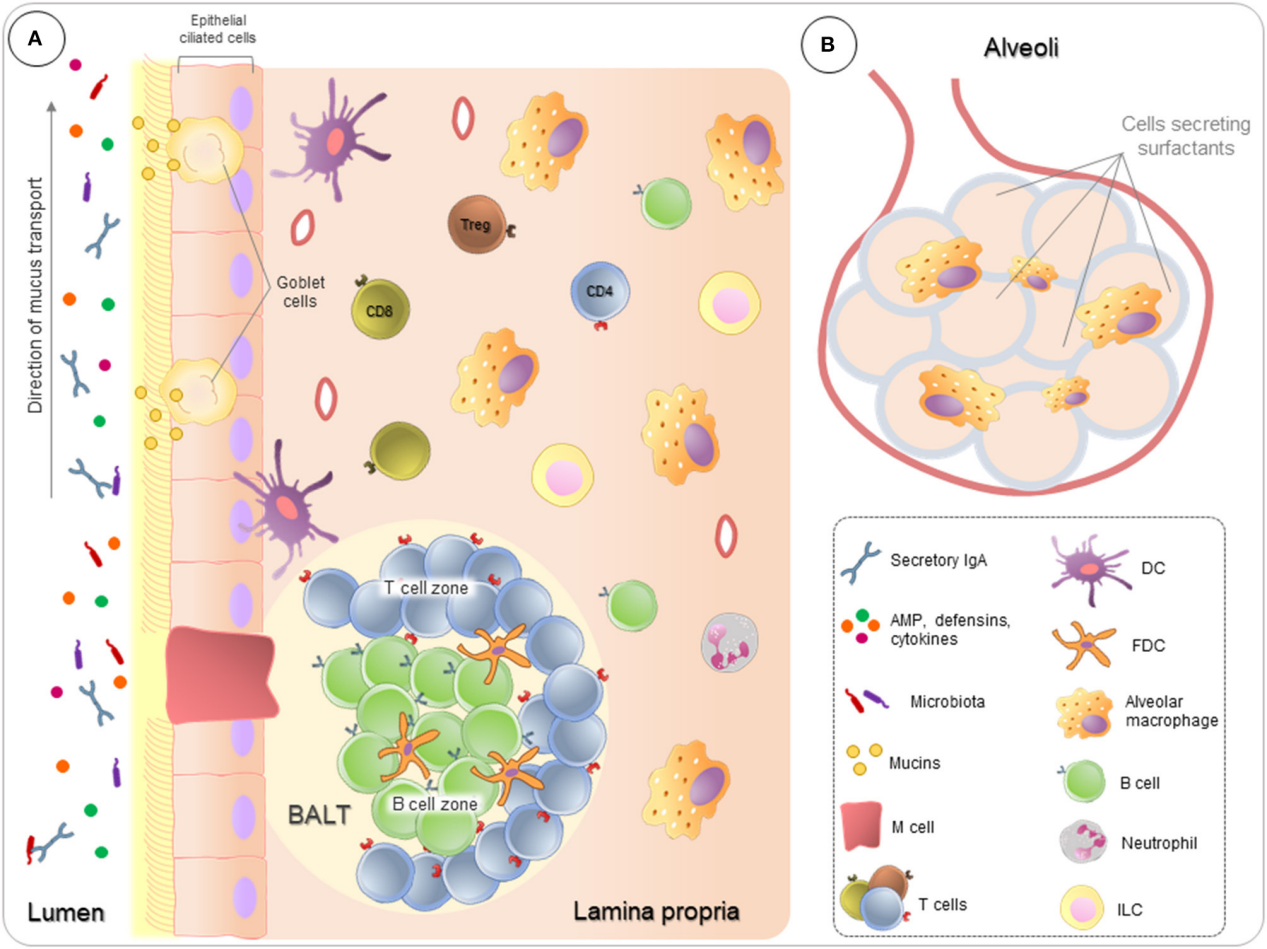
Pulmonary immunity overview. Lungs are in constant contact with many suspended substances, which are relatively harmless, such as pollutants, microbiota, and allergens. **(A)** Ciliated pseudostratified columnar epithelium covers all respiratory tract, providing a pathway for movement and conditioning of the air entering the lungs, as well as, controls the actions of the mucociliary escalator directing the particles to outside the lung. In association, Goblet cells produce mucins to create a protective layer of mucus, forming a first barrier of defense. Airway fluids also contain antimicrobial peptides (AMP), defensins, cytokines, and antibodies, mainly secretory IgA. To ensure the homeostasis, in the lamina propria, immune cells act both to identify and respond to sterile threats and to control the inflammatory process, preventing that inflammation from compromising lung function. In addition, follicular regions rich in T and B lymphocytes (BALT–bronchus-associated lymphoid tissue) play a crucial role in fighting against infections. **(B)** Alveolus are in broadly interaction with the external environment, where lung cells secrete surfactants proteins and alveolar macrophages are highly phagocytic.

The airway mucosa also contains PNECs and clusters of innervated cells called neuroepithelial bodies (NEBs), which are referred to as a set of “pulmonary neuroendocrine systems” (3, 45). These cells secrete amines, for example, serotonin, and peptides, for example, bombesin. PNECs play a role in the growth and differentiation of lung cells, while NEBs degranulate in the presence of hypoxia, acting as hypoxia-sensitive chemoreceptors (3). Immune cells and their organization in the lung are illustrated in Figure 1.

Although the lungs have mainly respiratory functions, there are descriptions of their role in non-respiratory processes. The lung has a very stretchable vasculature, which allows it to deal with variations in venous return, especially during postural changes, exercises and increased intravascular volume. When there is an increase in cardiac output, underperfused areas of the pulmonary vasculature are recruited to accommodate the increase in blood flow and prevent an increase in pulmonary arterial pressures (3). The pulmonary endothelium is also a source of fibrinolysin activator, which converts plasminogen to fibrinolysin. The lung, therefore, has an efficient fibrinolytic system, capable of smoothing clots in the pulmonary circulation (46).

In addition, the lungs are also an essential site for the degradation and inactivation of chemical mediators, playing a role in the biotransformation and detoxification of inhaled substances (3). The lungs express enzymes related to the metabolism of xenobiotics, the main enzymes being involved in the cytochrome P450 family and participating in oxidative metabolism, as well as in the metabolic bioactivation of many organic toxins, including pro-carcinogens (47). An important role of the lungs can therefore be to act as a buffer binding to xenobiotics, preventing an acute increase in systemic concentrations, as well as playing a role in the biotransformation of inhaled substances (47).

All these data showed the complexity of the mechanisms and interactions between immune cells, pulmonary epithelium cells and external agents (microbiota, innocuous agents, pathogens), for the maintenance of lung health and its respiratory activity.

## Nutrient Interference in Homeostasis, Lung Infections and Inflammatory Lung Diseases

Homeostasis can be defined as the stability of a complex system via internal mechanisms of self-regulation resilient to external disturbances (48). Considering that the lungs are chronically exposed to various pathogenic or non-pathogenic environmental antigens, maintaining a network of cells residing in the tissue that continuously monitors the external environment and instructs tolerance to innocuous inhaled particles is of paramount importance to ensure pulmonary homeostasis (6).

In this sense, diet and nutrition are becoming increasingly recognized as modifiable contributors to lung health, thus being a central parameter that governs the systemic immune system, homeostasis and pulmonary inflammation. Different dietary components can have direct effects on lung health or be used as sources of energy for immune function. In fact, their resulting metabolites can be potent immune modulators (23).

However, interruption of homeostasis in response to chronic inflammation, constant levels of harmful inhalants, senescence, genetic aspects, and/or infections can lead to morphological and functional changes in the lungs (49).

COPD is a common respiratory disease characterized by functional and structural changes generated, especially, by inhaling harmful particles (49). It has been considered a disease due to the chronic inflammation leading to remodeling of the extracellular matrix, in which the extent of the inflammation is related to the degree of airflow obstruction (50). In addition to inflammation, the imbalance between proteases and antiproteases and oxidative stress are processes involved in the pathogenesis of COPD (51).

The activation of PRRs by damage-associated molecular patterns (DAMPs), which are released after tissue damage, results in the synthesis of inflammatory cytokines. One of the possible mechanisms of cytokine production involved in the pathogenesis of COPD is dependent on caspase 1 and the formation of the P3 inflammasome of the nucleotide-binding oligomerization domain (NLR) (49). The NLRP3 inflammasome leads to the secretion of IL-1β and IL-18, which activate neutrophils, macrophages, Th1 and Th17 lymphocytes, leading to inflammation of the airways (52). Neutrophils are strongly involved in inflammation, and high levels of sputum neutrophils are associated with the severity of COPD (53). In addition, the reduced phagocytic function of macrophages influences the reduction of neutrophil apoptosis, which can generate secondary necrosis (54).

The oxidative load is also increased in COPD. The release of reactive oxygen and nitrogen species released by inflammatory cells promotes oxidative stress, which may lead to inactivation of antiproteases or stimulation of mucus production. It can also increase the activation of transcription factors (such as nuclear factor κB) and therefore, the gene expression of proinflammatory mediators (51).

Another inflammatory respiratory disease is asthma, which is a common chronic airway disorder characterized by variable and recurrent symptoms, airflow obstruction, bronchial hyperresponsiveness and underlying inflammation. Atopy, or the genetic predisposition to develop specific IgE antibodies against environmental allergens, is the strongest risk factor for the development of asthma. Although asthma has been viewed as a reversible disease, evidence indicates that permanent structural changes in the airway are typically seen and include subbasement membrane fibrosis, smooth muscle hyperplasia, new vessel formation, and glandular hyperplasia (55). In this context, long-term asthma is associated with an accelerated decline in lung function. Although lower than that observed in individuals with COPD, it is also an indication of structural and possibly permanent changes in the airways (55).

The inflammatory response in the airways of patients with asthma involves an interaction of the respiratory epithelium, innate immune system and adaptive immunity that initiates and leads to a chronic inflammatory response (56).

Prospective analyses since birth show that most asthma development occurs in early childhood and has an allergic component. Individuals with allergic asthma have eosinophilic inflammation in the lungs, as well as increased mediators related to the adaptive response of the Th2 subset, such as IL-4, IL-5 and IL-13, and serum IgE elevation. The Th17 subset may also play a role in asthma by mediating neutrophilic inflammation (57). The decrease in the suppressive activity of Treg cells (IL-10- and TGF-β-producing cells) represents additional mechanisms that contribute to asthma, perhaps partly distorting the system toward an increased Th2 response (58).

Although respiratory diseases extend throughout the course of life, the onset of asthma occurs in childhood, while COPD is commonly associated with groups in advanced age. In addition, there are significant changes in immunity associated with aging, with an increased risk of lung disease in the elderly (23).

The respiratory tract is constantly exposed to the external environment and must be prepared to respond to pathogens. Although an effective immune response to eliminate viral pathogens is essential, a prolonged or exacerbated response can cause damage to the respiratory tract. Thus, the antiviral immune response represents a balancing act between virus elimination and immune-mediated lung injury (59).

Respiratory viruses, including influenza viruses, respiratory syncytial viruses, coronaviruses, rhinoviruses, parainfluenza viruses, adenoviruses and human metapneumoviruses, among others, can lead to serious diseases in the respiratory system, such as pneumonia and/or exacerbation of asthma and chronic obstruction of lung disease (60).

Respiratory epithelial cells are usually the first cell type to be infected. The PRRs expressed in these cells recognize viral pathogen-associated molecular patterns (PAMPs) triggering the production and release of type I and III interferons and other proinflammatory mediators, such as cytokines, chemokines and antimicrobial peptides, which initiate the innate and adaptive immune response. Thus, the degree of activation of PRR influences the degree of recruitment of immune cells and release of proinflammatory mediators and, subsequently, any resulting immunopathology (59).

Type I IFNs, produced under the stimulation of viral recognition, bind to interferon alpha and beta receptor (IFNAR) expressed ubiquitously by activating the JAK/STAT pathway and inducing the expression of interferon-stimulated genes (ISGs) that act in limiting the spread and infection of the virus (61). Type I IFNs also directly promote lymphocyte functional activity by stimulating IFN-γ secretion, which in turn activates macrophages and phagocytosis, increases the presentation of antigens through DCs and limits viral replication. Type I IFNs also increase the cytotoxic activity of T cells and Natural Killer (NK) cells and promote the humoral response (62). As a consequence of widespread effects on host immune responses, IFNs can facilitate inflammation and injury to the lungs in an indirect manner during acute viral infection (59).

**Figure 2:**
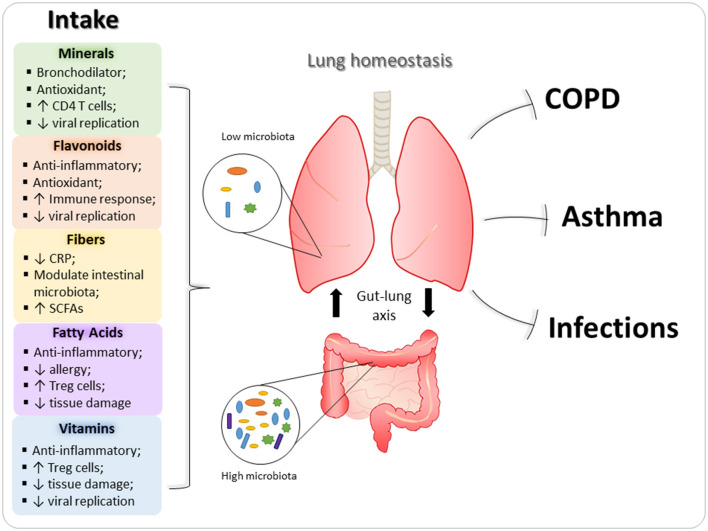
Immunomodulatory properties of nutrients in lungs. Nutrients are able to ameliorate the development and severity of pulmonary diseases, since they can act on several immune cells and modulate immune response in inflammatory processes. In this context, minerals, flavonoids, vitamins, and fatty acids reduce the expression of inflammatory mediators (such as cytokines and chemokines), as well as, have antioxidant effect, decreasing the deleterious effects of asthma and chronic obstructive pulmonary disease (COPD) in the lungs. In addition, fibers and fatty acids also can modulate intestinal microbiota, which contribute to lung homeostasis through gut-lung axis. Regarding pulmonary viral infections, vitamins, and flavonoids are the main dietary components with antiviral action.

Diet and nutrition can be important, modifiable, risk factors for the development, progression and management of obstructive pulmonary diseases, such as asthma, COPD, and pulmonary viral infections. Dietary factors with a potential protective role in the oxidative process and inflammatory response have been implicated in the genesis, evolution or protection against these diseases (12). Below, the main nutrients that can influence homeostasis and lung diseases are described (Figure 2). In addition, immunomodulatory role of nutrients in these pulmonary diseases are exemplified in Table 1.

**Table 1:** Immunomodulatory proprieties of nutrients in lung inflammatory diseases.

**Pathology**	**Immunomodulatory role of nutrients**	**References**
Asthma	Vitamins: • Vitamin A increases Treg cells and inhibits Th2 and Th17 cytokines in the lung; • Vitamin E reduces symptoms and decrease Th2 cytokines local and systemic ways	(63–65)
	Minerals: • Magnesium has bronchodilator effects and improve lung function; • Selenium protects airways membranes from oxidative damage	(66–68)
	Flavonoids • Suppress eosinophil infiltration and inhibit degranulation of mast cells and basophils; • Decrease fibrotic factors, edema and attenuate hyperresponsiveness	(69–73)
	Fibers: • Can modify gut microbiota increasing SCFAs, which regulate neutrophils and decrease allergic process	(74)
	• Fatty acids: • ω-3 reduces asthma score, increase acetylcholine and prevented allergen-induced reactions; • SCFAs protect from allergic lung inflammation and induces Treg cells inlung	(75–77)
COPD	Vitamins: • Vitamin D deficiency is correlated with impaired control of inflammation, associated with NF-κB and AP-1 signaling activation	(78)
	Minerals: • Severe COPD is associated with lower serumzinc	(79)
	Fibers: • Reduce risk of COPD, since reduce systemic inflammations and C-reactive protein levels	(74)
Viral infections	Vitamins: • Vitamin A decreases the viral load and inflammatory cytokines, increases IFN-β, inhibits replication, and improves antibody response; • Vitamin C increases IFN-γ production by NK cells and cellular antiviral response; • Vitamin D induces antimicrobial peptides with antiviral activity and high levels of the iNF-κB inhibitor and decreases inflammatory cytokines	(80–86)
	Minerals: • Zinc induces Treg cells and the damping of Th17 and Th9 cells, increases CD4 T cells, contributes to adequate BCR antibody response, as well as inhibits RNA polymerase (necessary for the replication of RNA viruses)	(87–90)
	Flavonoids: • Improve antibody response, cytotoxic activity and increase Treg cells; • Reduces viral replication and lung injury during infection	(69, 91–95)
	Fibers: • Increases SCFAs synthesis leading to maturation and destiny of immune cells	(23)
	• Fatty acids: • SCFAs promotes recruitment of immune cells to airways and protect against infections • Acetate increases production of IFN-β and protects from respiratory infection through receptor for acetate GPR43	(74, 96, 97)

*SCFAs, short-chain fatty acids; ω-3, omega-3*.

### Vitamins

Vitamins are micronutrients available in several kinds of foods and can be of animal or vegetable origin. In addition to their nutritional role, they also participate in immunity and homeostasis of the mucosa, such as the intestinal and pulmonary mucosa. Regarding lung health and homeostasis, vitamins A, C, and D can be considered the most important, not only for their anti-inflammatory action but also for participating in the immune response against pathogens, as we describe below.

Several studies on vitamin A have shown that the active metabolite retinoic acid (RA) has a fundamental role in the maintenance and modulation of the immune response and the homeostasis of epithelial tissues and mucosa and is important in the control of inflammatory diseases (98).

In clinical studies, vitamin A deficiency can be correlated with asthma, since serum retinoid concentrations are significantly lower in patients with asthma than in healthy control subjects, mainly in patients with severe asthma (99, 100). In an experimental asthma model, vitamin A deficiency in mice also worsened the inflammatory condition by increasing the Th2 cytokines IL-5 and IL-13 and pulmonary inflammation (101). However, the administration of RA increases Treg cells in the lungs, attenuating inflammation (63). Supplementation with RA also promotes downregulation of the GATA3 and RORγt transcription factors, inhibiting Th2 and Th17 cytokines in the lungs (64) highlighting the importance of adequate intake of vitamin A for asthma.

Vitamin A also plays an important role in the humoral response and antiviral mechanisms of the immune response. For example, RA is essential to the production of IgA antibodies (102) and RA deficiency associated with zinc deficiency leads to a decrease in serum IgA, promoting damage to humoral immunity (103). In addition, because of vitamin A deficiency, mice infected with influenza present failures in CD4^+^ T-cell recruitment and B-cell organization into lymphoid structures in the lungs and increasing mortality rates after bacterial coinfection (104).

Moreover, RA demonstrated the ability to decrease the viral load of *Morbillivirus* in infected mice (80). This decrease was possible due to the mechanism that induces RIG-1 expression, promoting the production of type I IFNs by increasing the recognition of viral dsRNA by immune cells. Another study showed that treatment with retinol or RA in mice infected with acute gastroenteritis virus (*Norovirus*) increased the production of IFN-β, inhibiting norovirus replication (81).

In the context of obesity, vitamin A supplementation significantly improved vitamin A levels in the lungs of diet-induced obese mice, decreased inflammatory cytokines in the blood and improved antibody responses after vaccination against the influenza A (H1N1) virus, thereby promoting a reduction in viral loads post challenge (82). These data suggest that vitamin A has a strong impact on the vaccine response.

Vitamin C is another important micronutrient for the immune response in the lungs. A trial study with 197 elderly patients with pneumonia has shown that treatment with two doses of vitamin C was able to reduce severity and mortality (105). Moreover, another antiviral effect of vitamin C could be seen in a study with H1N1-infected mice. In this study, supplementation with vitamin C increased IFN-γ production by NK cells and reduced the viral infection and lung inflammation induced by H1N1 infection (83). Similar results have been shown in peripheral blood mononuclear cell (PBMC) culture with increases in CD25 and CD69 expression in T and NK cells, promoting the activation of the cellular antiviral response (83).

Vitamin D is important for lung health since birth, even in the absence of infections. A recent study showed a correlation between vitamin D deficiency and respiratory distress syndrome in premature infants (RDS) (106). In this same study, it was also shown that patients with higher 25(OH)D levels can be preventive for the development of RDS. RDS presents as the main characteristic of pathological surfactant deficiency and pulmonary immaturity, demonstrating the role of vitamin D in promoting lung maturity.

Vitamin D also has great potential in modulating the immune response against respiratory viruses (107). For example, vitamin D regulates the expression of antimicrobial peptides LL-37 and β-defensin 2 (108, 109), both with antiviral activity against respiratory syncytial virus (RSV) (84, 85). These antimicrobial peptides block viral cellular entry, inhibiting the production of new infectious particles and consequently, diminishing the spread of infection, and virus-induced epithelial cell death (110).

The antiviral effect of vitamin D was also described in RSV infection in the first year of life, since vitamin D deficiency has a positive correlation with RSV infection (111). In addition to antiviral effects, vitamin D also presents anti-inflammatory effects during viral infections. *In vitro* studies with primary human tracheobronchial epithelium (hTBE) pretreated with the active form of vitamin D, 1,25-dihydroxycholecalciferol [1,25(OH)2], showed less proinflammatory cytokine production after RSV infections due to high levels of the NF-κB inhibitor IκBα and IκBα induced by vitamin D pretreatment without affecting the antiviral response (86). Similar results have been shown in a treated human alveolar epithelial-cell line with 1,25(OH)2 D3 before or after influenza A (H1N1) exposure, with a decrease in proinflammatory cytokines and virus-induced cell death but without an effect on viral clearance (112).

The anti-inflammatory effect of vitamin D can also be seen in COPD patients. Recently, it has been shown that COPD patients present low serum levels of 25(OH)D and high serum levels of proinflammatory cytokines compared to healthy individuals. Vitamin D deficiency was more robust in patients with grade 4 COPD (78). These patients also presented NF-κB and activator protein 1 (AP-1) signaling activation and a decrease in the NF-κB inhibitor IκBα and IκBα. All these data showed the importance of vitamin D in controlling this inflammation.

Regarding vitamin E, there are few reports that describe its immunoregulatory role in the lungs; however, in a recent study with an experimental model of asthma and allergic rhinitis, it was demonstrated that vitamin E reduced the symptoms of the pathology due to its anti-inflammatory action (65). In this study, mice treated with vitamin E showed improvement in the pulmonary inflammatory condition, with a decrease in the presence of serum Th2 cytokines and in bronchoalveolar lavage; in addition to a decrease in constriction and mucus secretion in the mice, especially in combination with selenium.

Together, these data show the importance of adequate consumption of vitamins for the maintenance of lung health.

### Minerals

Minerals are inorganic substances present in food, such as magnesium, selenium, and zinc, that play an important role in cellular metabolism and the immune response. Magnesium works with calcium (Ca^2+^) to affect neuromuscular transmission and activity and can block or compete with calcium in voltage-dependent channels operated by receptors or leaks, resulting in translocation of intracellular Ca^2+^. With this, magnesium inhibits the release of Ca^2+^ from the sarcoplasmic reticulum, modulating smooth-muscle contractions and the rate of relaxation (113, 114). Dietary magnesium has shown beneficial bronchodilator effects in asthma (113) as well as in severe asthma exacerbations, and a single dose of intravenous magnesium sulfate (MgSO^4^) was able to reduce hospitalizations and improve lung function (66). On the other hand, the deficiency in magnesium consumption in asthmatic individuals was related to negative actions in the bronchial smooth muscle (12, 115).

However, it was also described in a clinical trial with asthmatic individuals that supplementation with magnesium administered together with vitamin C did not demonstrate any clinical benefit in lung function, symptoms or the possibility of decreasing the dose of steroids (116).

Selenium acts as an antioxidant and, when interacting with other nutrients, such as vitamin E, protects cells against oxidative stress. Selenium is an essential component of the enzyme glutathione peroxidase (GSH-Px), which reduces hydrogen peroxide and other organic peroxides to harmless substances. By detoxifying peroxides, GSH-Px prevents peroxidation and subsequent instability of cell membranes. It has been proposed that selenium, as a component of GSH-Px, can protect membranes in asthmatic airways from peroxide-induced damage (67).

In this context, previous studies have shown that peripheral blood and platelet GSH-Px activity is reduced in sensitive asthmatic patients (117) and case-control studies have reported lower levels of selenium in the blood of individuals with asthma compared to controls, with selenium being negatively associated with asthma (118, 119). However, a study with children did not find a relationship between the levels or intake of selenium and results related to asthma (120). Studies with adult asthmatic subjects who were on inhaled-steroid use for 24 weeks did not reveal any clinical benefit from selenium supplementation (119, 121).

Zinc is essential for the synthesis of DNA and is an enzymatic cofactor that participates in various physiological and metabolic functions in the body. It is also known to induce the production of metallothionein, which is rich in cysteine and is, therefore, a potent OH-radical scavenger (122).

In relation to control subjects, a study showed that COPD patients had lower serum zinc concentrations, and this reduction was even more pronounced in patients with COPD grade III (severe COPD) compared to those with milder disease (grades I and II) (79). Low plasma zinc content has been associated with respiratory tract infections in children, while zinc supplementation has been associated with a reduction in the incidence of pneumonia in children without vitamin A deficiency (13, 123). Zinc supplementation improves immune functions, including reduced skin hypersensitivity and an increased number of TCD4 cells (87, 124).

The altered proportion of Th1 and Th2 cells in favor of allergic reactions induced by Th2 cells is a consequence of zinc deficiency; therefore, zinc plays an important role in the proper differentiation of T cells. In addition, tolerogenic immunoreaction is triggered by changes in intracellular zinc levels due to the induction of Treg cells and the damping of proinflammatory Th17 and Th9 cells (88).

In experimental models, zinc deficiency has been shown to impair cellular and humoral immune function (125, 126), whereas, the zinc transporter 10 (ZIP10) is necessary for adequate antibody responses after B-cell receptor (BCR) activation (89).

In the context of viral infections, it has been reported that zinc is able to inhibit the RNA polymerase necessary for the replication of RNA viruses, indicating that zinc may play an essential role in the defense of the host against RNA viruses (90). The replication of influenza virus was inhibited *in vitro* by the zinc ionophore pyrrolidine dithiocarbamate (127).

In mechanistic terms, the strong correlation between homeostatic iron concentrations and the presence of oxygen in the lungs is evident, where both systems must be adequately controlled for full lung function. Oxygen to be transported efficiently by erythrocytes depends on the presence of hemoglobin, a protein capable of binding oxygen through its central iron atom (128).

In addition to participation in hemoglobin synthesis, iron is of great importance for other essential metabolic processes, such as DNA repair, transcription and energy production in mitochondria. However, free iron is highly reactive and potentially toxic and is able to catalyze the production of reactive oxygen species (ROS) and damage lipids, nucleic acids, and proteins, causing tissue damage (129). For this reason, iron is mostly linked to protein groups to neutralize its reactivity.

Therefore, like any other cell, lung cells must acquire adequate amounts of iron to supply metabolic needs and to ensure lung function and survival. In parallel, lung cells must avoid excess iron, oxidative stress and resulting injuries that can impair lung function (130).

### Flavonoids

Growing evidence suggests that natural polyphenols, particularly flavonoids, can ameliorate the inflammatory process (131). Flavonoids are polyphenolic compounds broadly present in plants (132). According to their structures and the hydroxylation and glycosylation patterns of benzene rings, flavonoids can be present in different subclasses, which include flavanols, flavanones, flavones, isoflavones, flavonols, and anthocyanidins (133). These bioactive compounds are particularly abundant in the human diet of fruits, vegetables, tea, red wine, chocolate, and coffee (134). However, their considerable structural diversity and *in vivo* bioavailability allow them to modulate different signaling pathways (135).

The immunomodulatory properties of flavonoids are associated with the inhibition of protein kinases, enzymes involved in arachidonic acid metabolism and the regulation of key signaling pathways, such as NF-κB and nuclear-related factor 2 (Nrf2) (136–140). Additionally, they have antioxidant effects due to their scavenging activity for reactive oxygen or nitrogen species and by a reduction in oxidative stress (138, 141). Some flavonoids also exert anti-inflammatory effects by blocking the NLRP3 inflammasome, inhibiting proinflammatory cytokine production, and downregulating chemokines (142–144).

Flavonoids are reported to possess a wide variety of biological activities on immune cells, modulating their activation, differentiation and proliferation, and they can act on neutrophils, T cells, NK cells, DCs, and macrophages by reducing the expression of proteins and receptors (69, 70, 138). In this context, eosinophils, neutrophils, mast cells and basophils are also affected by flavonoids, which inhibit degranulation and decrease the release of histamine and other mediators (71, 72, 145). In addition, the improvement of the immune response is associated with antibody production, cytotoxic activity, and enhancement in regulatory T cells (91).

Some studies point to the antiviral properties of flavonoids against a wide range of DNA and RNA viruses. For example, apigenin (flavone) is active against picornavirus (RNA virus), inhibiting viral activity (92); catechin (flavanol) reduces the replication cycle of the hepatitis B virus, herpes simplex and adenovirus (93); naringenin (flavanone) has antiviral activity against dengue, Zika, hepatitis C, chikungunya, yellow fever, and human immunodeficiency virus (69). In addition, flavonoids were found to reduce lung injury and the inflammatory response during influenza H1N1 infection in a mouse model (94). Recently, flavonoids have also been proposed against coronavirus infection (69, 95).

There are strong evidences that concerns the role of flavonoids in several pulmonary diseases through decreased release of inflammatory mediators, fibrotic factors, and edema, and the attenuation of Th17 inflammation and suppression of airway hyperresponsiveness (69, 72, 73). Furthermore, flavonoid supplementation is also effective in reducing the incidence of upper respiratory tract infections (146).

In general, chronic diseases are caused by chronic inflammation; therefore, flavonoids have been proposed as potentially useful treatments for inflammatory diseases.

### Fibers

Dietary fibers are defined as the edible parts of plants or analogous carbohydrates. They are resistant to digestion and absorption in the human small intestine and are completely or partially fermented in the large intestine. Dietary fiber includes polysaccharides, oligosaccharides, lignin and associated plant substances. Studies indicate that fiber intake can reduce the risk of COPD due its anti-inflammatory effect, since systemic inflammation is an important feature of COPD (147).

Increased dietary fiber intake has been linked to reduced systemic inflammation and C-reactive protein (CRP) levels (74). Considering that CRP is a marker of systemic inflammation activated by the innate immune system and a possible molecule associated with vascular disease (148), it is possible that its action is related to lung damage. High-fiber diets were also able to reduce mortality from infectious or inflammatory diseases (50% reduction), respiratory diseases (60% reduction), and smoking-related cancers (25% reduction) (149).

Dietary fibers can also modify the intestinal microbiota, especially interfering with the ratio between Firmicutes and Bacteroidetes. As a result, there is an increase in short-chain fatty acids that are derived from the fermentation of dietary fibers. These fatty acids have relevant protection in the regulation of neutrophils, lung function and COPD, and epithelial protection against infection (150).

According to the European Food Safety Authority, the current recommendation for dietary fiber intake is 25 g/day (151).

### Fatty Acids

Most of the lipid mediators that regulate inflammation are metabolites from omega-6 (ω-6) or omega-3 (ω-3) fatty acids, including arachidonic acid, linoleic acid, eicosapentaenoic acid and docosahexaenoic acid. ω-3 and ω-6 are considered essential fatty acids, as the body is not able to produce them, and their acquisition through diet is necessary (152). Most vegetable oils are significant sources of ω-6, while cold-water marine fish are the main sources of ω-3 (153).

Generally, ω-6 fatty acids are proinflammatory, and ω-3 fatty acids are anti-inflammatory (154). Epidemiological data describe that populations with a higher intake of ω-6 fatty acids have a higher prevalence of asthma in relation to those that consume smaller amounts of ω-6 and with a higher intake of ω-3 fatty acids, considering that ω-3 produces ecosystems that are less proinflammatory than those derived from ω-6 (154, 155).

Food supplementation with fish oil rich in ω-3 fatty acids for 10 months was able to reduce asthma scores and increase acetylcholine thresholds in children with bronchial asthma (75). In other studies, the administration of fish oil prevented only allergen-induced late asthmatic reactions and had no effect on immediate reactions (76). In a murine model, ω-3 supplementation improved survival, reduced bacterial invasion into the blood and lungs, and decreased overall lung tissue inflammation and cell death compared to ω-6-supplemented diets (156).

Another fatty acid described in lung protection is short-chain fatty acids (SCFAs). SCFAs are derived from the fermentation of fibers by means of intestinal bacteria and are essential to regulate a wide variety of processes in the gastrointestinal tract, but they are also potent mediators of the function, maturation, and destiny of immune cells (23). Oral application of SCFAs to mice during pregnancy and weaning protected the offspring from allergic lung inflammation, potentially inducing Tregs in the offspring's lungs (77).

SCFAs such as acetate and butyrate, in addition to their anti-inflammatory activity (157), also play an important role in infectious diseases. Galvão et al. showed that the absence of the receptor for acetate GPR43 increased susceptibility to *Klebsiella pneumoniae* infection, with uncontrolled proliferation of bacteria and an inflammatory response. On the other hand, treatment with acetate was efficient for protection during bacterial lung infection (96). Against RSV infection, acetate also protects host mice through GPR43 via another mechanism. In RSV infection, the antiviral effect is caused by increasing the expression of interferon-stimulated genes in the lungs, leading to the production of IFN-β cytokine (97).

In a murine model, it was also seen that SCFAs were able to promote the recruitment of neutrophils into the airways and to protect against infection from the influenza virus (74).

## Gut-Lung Axis and Pulmonary Immune Response

The microbiota is a constituted by the microbial commensal communities includes bacteria, fungi, viruses and protozoa that reside in different tissues, especially in gut (158). Its functions range from breaking down complex dietary polysaccharides to competing with pathogens and modulating the mucosa and the development of the immune system, both locally and systematically (159, 160). However, some studies demonstrate that the nutritional status, since childhood, impacts not only in the immune response and homeostasis but also in the intestinal microbiota, evidencing another way in which nutrition can impact mucosal immunity (161).

Regarding pulmonary health and maintenance of homeostasis, experimental evidence has highlighted a cross between the intestinal microbiota and the lungs, called the intestine-lung axis (5). Thus, the intestinal microbiota, influenced by nutrition, plays an important role in the immune responses developed during infections and inflammatory lung diseases. Below we will describe some aspects regarding the intestine-lung axis in two subsections: gut and airway microbiota.

### Gut Microbiota and Gut-Lung Axis

With a bacterial load on the order of 10^14^ bacteria (162) the intestine is the most densely colonized surface of the human body, home to between 100,000 and 100 billion bacteria per ml of luminal content (163). The microorganisms present in the intestinal microbiota act as a source of PAMPs that, when recognized by PRRs as TLRs, are in direct contact with the intestinal lumen and promote the proliferation of epithelial cells, expression of antimicrobial peptides and secretion of IgA (164). In addition, the gut microbiota can influence host immunity by inducing the release of anti-inflammatory (IL-10), and proinflammatory (IFN-γ, IL-17, IL-6, and IL-12) cytokines (165), releasing metabolites (166–168), and controlling the function of phagocytes, including DCs (169).

The diversity of the intestinal microbiome has genetically determined variations, but it is also influenced by environmental factors, such as lifestyle and diet (170). For example, variations in the intake of resistant starch or non-starch polysaccharides have been reported to alter specific bacterial-rate levels, such as *Ruminococcus bromii* and *Eubacterium rectal* (171), just as the composition of the intestinal microbiota in breastfed babies (superior bifidobacteria, lactobacilli, staphylococci, and streptococci) differs considerably from formula-fed babies *(Bacteroides, Clostridia*, and *Proteobacteria)* (172). Regarding obesity, the high consumption of ultra-processed foods, in addition to causing a state of micronutrient deficiencies, may be related to dysbiosis, demonstrating the importance of diet in maintaining a healthy microbiota (173).

Dysbiosis in gut microbiota can impair immune responses and pulmonary homeostasis. In this context, studies in germ-free and antibiotic-treated mice have contributed to the understanding of the relationship between intestinal microbiota and local and systemic homeostasis (174). Fecal transplantation in these animals, thereby reconstituting their microbiota, was able to restore intestinal immunity, influence the development of the mucosal systemic immune system and protect against bacterial and viral infections (175).

An experimental model of dysbiosis induced by antibiotic ingestion decreased effector and memory T cell populations in mice infected by *Mycobacterium tuberculosis* (176), since dysbiosis affected the activation of innate receptor macrophage inducible C-type lectin (mincle) of lung DCs. After the microbiota is restored, DC's ability to activate T cells is also restored.

Dysbiosis can also impair the immune response against H1N1 infection (177), since intact microbiota composition is critical to the generation of virus-specific CD4^+^ and CD8^+^ T cells and antibody responses following infection in an experimental model. In addition, dysbiosis present in obesity can also be related to changes in the immune response during lung infections (173). On the other hand, promote a healthy gut microbiota is important against pulmonary infections. Study with microbiota transplantation in gut microbiota-depleted mice infected intranasally with *S. pneumoniae*, showed that microbiota was able to control bacterial dissemination and inflammation (178).

The transplantation of isolated group of host-adapted commensal organisms, such as Segmented filamentous bacteria (SFB), also play an important role in lung infections without the need for transplantation of all components of the gut microbiota. In this context, a study with immunodeficient Rag^−/−^ mice infected with *S. pneumoniae* showed that the transplantation of SFB influenced lung protection, not for controlling bacterial infection, but for regulating innate immunity (179). In this study, the SFB promoted a shift in lung neutrophil phenotype from inflammatory neutrophils to pro-resolution neutrophils with low CD18 and high CD62L reducing, this way, the severe tissue damage caused by inflammatory neutrophils. So, the gut microbiota can also act by decreasing the inflammatory response, reducing the tissue damage caused by the immune response.

Besides that, epidemiological studies have described a correlation between changes in the intestinal microbiota and susceptibility to the development of airway allergies. A reduction in the microbial variety in the intestine during childhood has been shown to increase the risk of developing asthma (180) and the use of broad-spectrum antibiotics may increase the predisposition to allergic airway diseases thus demonstrating the correlation in the intestine-lung axis.

### Airway Microbiota and Gut-Lung Axis

The respiratory tract, long considered sterile, is actually a dynamic, microbial ecosystem. Unlike the intestinal microbiota, the lower respiratory tract is one of the least populated sites by microorganisms in the human body, with an approximate number of 10–100 bacteria per 1,000 cells (181). Its composition is dependent on microbial colonization of the upper respiratory tract through salivary micro-inhalations, interactions with the host's immune system and environmental conditions such as pH and oxygen concentration (182).

The intestine and lungs develop in parallel after birth, with constant communication between these two compartments (158) with the bacterial phyla most common in the lower respiratory tract being the same as those in the intestine, mainly Firmicutes and Bacteroidetes, followed by Proteobacteria and Actinobacteria (183). On the other hand, the nasal microbiota is more similar to skin microbiota, with a prevalence of Firmicutes and Actinobacteria phyla (48, 184).

The mesenteric lymphatic system is an important communication route between the intestine and the lungs, through which intact bacteria, their fragments or metabolites can translocate through the intestinal barrier, reach the circulatory system, and modulate the lung's immune response (182). For example SCFAs, which are mainly synthesized through the fermentation of bacterial dietary fibers, act in the lungs as signaling molecules in cells presenting resident antigens, thereby reducing inflammatory and allergic responses (157).

However, the gut-lung cross-talk also can influence in the opposite way, when the lung infections or chronic inflammatory diseases induce alterations in gut microbiota. Chronic lung disorders, such as asthma and COPD, can exhibit not only dysbiosis in airway microbiota but also in gut microbiota with tissue damage (185).

In addition, respiratory influenza infections in mice indirectly induce intestinal immune injury and gut dysbiosis promoting inflammation through the outgrowth of *Enterobacteriaceae* and the reduction of *Lactobacilli* and *Lactococci* (186). After gut dysbiosis mediated by IFN-γ produced by lung-CCR9^+^CD4^+^ T cells recruited into the small intestine, the population of Th17 cells increased promoting the tissue injury.

Still during mice influenza infection, changes in gut microbiota composition, reduce the acetate production and affect the bactericidal activity of alveolar macrophages (187) contributing to pulmonary pneumococcal superinfection. However, it has been shown that intranasal administration of *Lactobacillus casei* may be able to protect and mitigate the symptoms from influenza virus infection (188) in neonatal and infant mice infected. Intranasal *Bifidobacterium longum* administration also protects against viral-induced lung inflammation and injury in murine model of influenza virus infection (189). In this study, the reduced viral load was associated with reduced lung injury and IL-6 inflammatory cytokine, besides a shift from neutrophil to macrophage recruitment and increased levels of IFN-λ and surfactant protein.

The intranasal administration probiotics has also been used in inflammatory lung diseases. A study with 24 patients with chronic rhinosinusitis showed benefits with of *Lactococcus lactis W136 bacteria i*ntranasal irrigation after 14 days, with increase of the *Dolosigranulum pigrum*, a bacteria identified as potentially beneficial in the upper airways (190). Another study in a mouse model of allergic asthma reported that intranasal administration of probiotic *Lactobacillus rhamnosus GG* prevents the development of asthma due to decrease in bronchoalveolar lavage the eosinophils cells, lung IL-5 and 13 levels, and airway hyperreactivity (191).

This way, modifications in airway microbiota can contribute to protection against infections and inflammatory lung diseases.

Together, these data show the importance of gut-lung cross-talk in maintaining pulmonary mucosa homeostasis, as well as in the immune response against pathogens and the development of inflammatory diseases.

## Bioactive Compounds and Epigenetic Regulation in Lung Health

Epigenetics is the transcriptional regulation of gene expression carried out by chemical changes in DNA, such as methylation, acetylation, phosphorylation, and regulation by miRNAs (microRNA), which result in phenotypic changes without promoting changes in the DNA sequence (192, 193). Transcriptional changes by acetylation are mediated by histone deacetylases (HDACs) and histone acetyltransferases (HATs). The deacetylation of histone lysine residues mediated by HDACs makes chromatin transcriptionally repressive, interfering with gene expression by inhibiting the access of transcription factors (194, 195). HAT-mediated histone acetylation makes chromatin transcriptionally permissive, thus favoring the binding of transcription factors and other transcriptional coactivators (194, 195).

In addition to histones, HDACs have other protein substrates, such as NF-κB. Sirtuin I (SIRT1), a class III HDAC, in addition to acting on histones, also acts on NF-κB, promoting deacetylation of the p65 subunit. Therefore, SIRT1 acts to suppress the transcription of proinflammatory cytokines (196).

HDAC and HAT activity has already been identified in nuclear extracts from lung-tissue specimens. Moreover, it has been reported that patients with COPD have a progressive reduction in total HDAC activity, reflecting the severity of the disease (197). HDACs are key molecules in suppressing the production of proinflammatory cytokines; they are understood to be an important component that can act on lung health.

Bioactive compounds may play roles in the regulation of HDAC activity and histone acetylation (198). Bioactive compounds are extranutritional constituents that are usually present in food in small concentrations and provide health benefits beyond basic nutritional value (199). These bioactive molecules can have therapeutic potential by influencing energy intake, in addition to reducing the proinflammatory state, oxidative stress and metabolic disorders (200).

Epidemiological studies suggest that the increase in consumption of foods rich in bioactive compounds with antioxidant activity, such as vitamins, phytochemicals, and especially phenolic compounds, may represent an important factor in the reduction of several pathologies, such as cancer, heart disease, stroke, and Alzheimer's disease (201).

Resveratrol is a polyphenolic bioactive compound found in several plant species, including grapes and peanuts, and is able to positively regulate SIRT1 in human pulmonary alveolar epithelial cells, reduce the production of ROS and inhibit apoptosis in alveolar epithelial cells, thus reducing lung injury (202). It has also been reported that SIRT1 activates peroxisome proliferator-activated receptor-gamma coactivator (PGC)-1α, an important regulator of mitochondrial metabolism. Therefore, resveratrol can improve mitochondrial function, which is usually compromised in the lungs of patients with COPD (203). In the context of viral infections, *in vitro* studies have indicated that treatment with a SIRT1 antagonist (EX-527) generates an increase in the production of influenza viruses and human cytomegalovirus (HCMV) infection, while SIRT1 agonists promote a reduction in the production of viral particles (204). Studies have also indicated a potential role for SIRT1 in regulating inflammation during allergic asthma due to significant inhibition of IL-6 expression (205).

However, most of the proposed therapeutic activities of resveratrol have not yet been confirmed in clinical trials. There are reports that in healthy individuals, a single dose of resveratrol (100 mg) combined with muscadine grape extract polyphenols (75 mg) is able to suppress the oxidative and inflammatory response to stress (206). There is also evidence that the safe daily dosage of resveratrol for an individual weighing 70 kg is 450 mg/day (207). However, more studies are needed.

Another bioactive compound, diferuloylmethane, is also capable of inducing epigenetic regulation and contributing to lung health. Known as curcumin or turmeric from India, this compound has a pleiotropic role, interacting with several molecular targets, such as transcription factors, proteins and enzymes associated with epigenetic modulations (208). By acting in the regulation of histone protein acetylation/deacetylation processes, it promotes changes in chromatin structure, which can influence inflammatory responses. Curcumin is able to specifically inhibit p300/CB-HAT (209).

Therefore, it promotes the suppression of histone acetylation and simultaneously promotes active activation to HDAC2 deacetylation, canceling the interaction between NF-κB and DNA; thus, preventing inflammatory responses that may be harmful to lung tissue (210). Considering that corticosteroids recruit HDAC2 as one of the mechanisms of action, it has been suggested that its induction through curcumin may be an important therapeutic target (211).

Curcumin also has direct anti-inflammatory actions through the inhibition of inhibition of IκB kinase (IKK), which degrades κB, a molecule capable of degrading the inhibitory protein of the NF-κB complex (212). In the context of viral infections, it has been reported that curcumin can provide protection against acute lung injuries induced by H1N1 infection by limiting the expansion of immune cells and reducing the production of proinflammatory cytokines via NF-κB (213).

The safe dose of curcumin is 12 grams per day; however, there are few clinical studies that have shown that ingesting curcumin can have anti-inflammatory effects, considering that one of its main disadvantages is its low bioavailability and hydrophobic nature (208).

Another compound that can influence lung health includes catechins. Catechins are among the biologically active compounds present in *Camellia sinensis*, known as green tea, and they are tea's main antioxidant agent. The catechins contained in tea include epigallocatechin-3-gallate (EGCG), epicatechin-3-gallate (ECG), epigallocatechin (EGC), and epicatechin (EC) (214). Among these, EGCG is the catechin that most demonstrates therapeutic effects. EGCG is shown to be a specific inhibitor of HAT, thus influencing histone acetylation and promoting an anti-inflammatory effect by inhibiting the P300-mediated acetylation of NK-κB. Moreover, it is also able to prevent the binding of p300. Its anti-inflammatory effect is mediated by inhibition of p300-mediated acetylation with the NK-κB promoter (208). It is described that ingestion through catechin feeding is able to improve lung health and to reduce shortness of breath and sputum in COPD (215). The average daily intake of EGCG resulting from the consumption of green tea infusions varies from 90 to 300 mg/day (216). However, further studies are needed to evaluate the most safe and effective dosage.

## Perspectives About Nutrition and Covid-19

SARS-CoV-2 quickly spread around the world in 2020, and has been classified as a global pandemic by the World Health Organization (217). This virus is the etiologic agent of coronavirus disease 2019 (COVID-19) which presents, in general, mild, and moderate symptoms, but a more severe manifestation can cause acute respiratory syndrome, multiple organ dysfunction syndrome and can lead to death (218, 219). Indeed, a little over a year and a half after the first case of the disease, more than 202.1 million people have already been infected with more than 4.2 million deaths (11).

**Figure 3:**
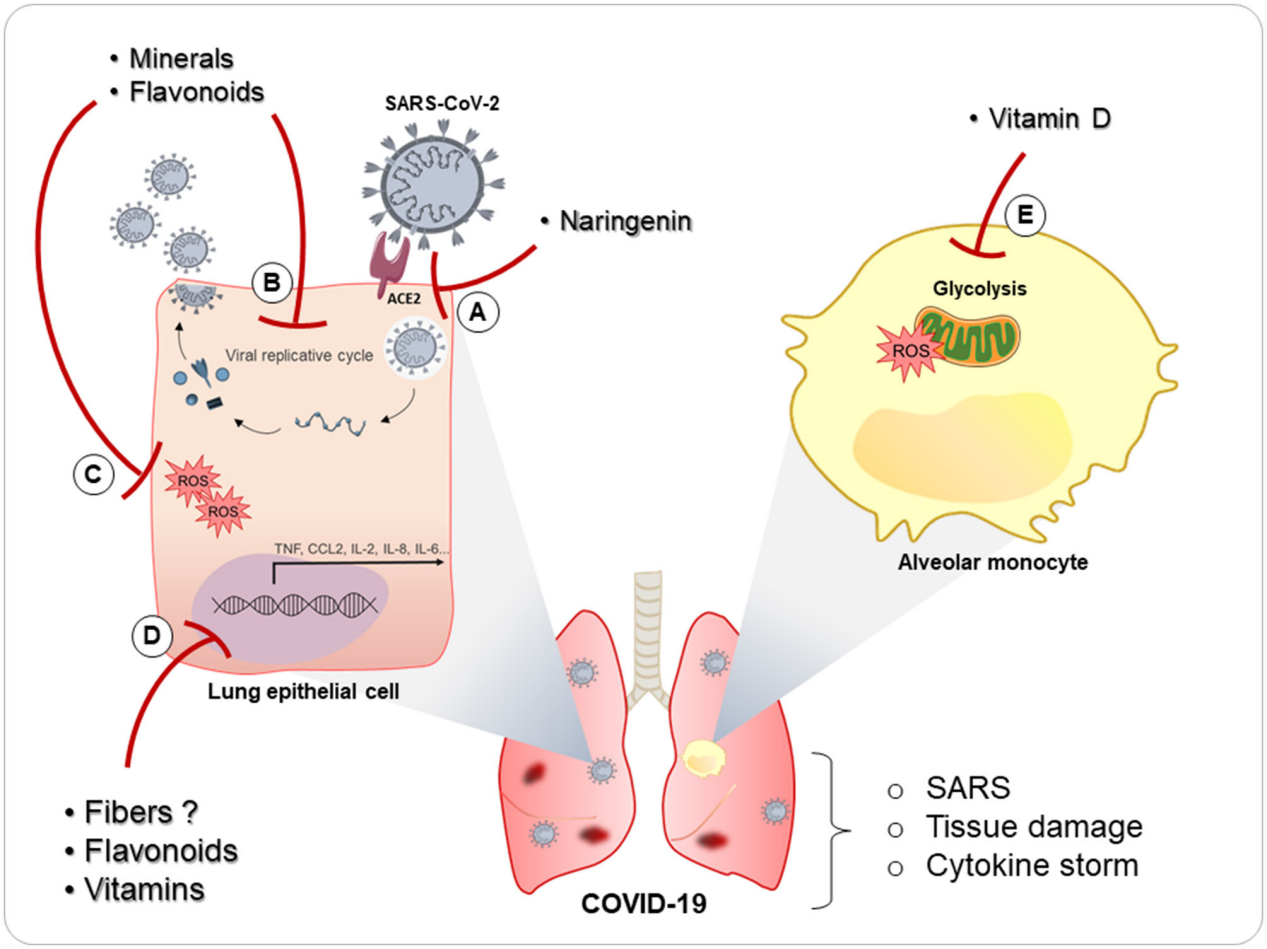
Possible role of nutrients in COVID-19 pulmonary pathophysiology. Some nutrients have been proposed during COVID-19. (A) Naringenin has been described as being able to inhibit infection SARS-CoV-2 infection. (B) Vitamins, minerals, and flavonoids can inhibit viral replication in many pulmonary infections, and naringenin already demonstrated ability to decrease viral replication of SARS-CoV-2. (C) These nutrients also have antioxidant role (inhibition of reactive oxygen species—ROS) and (D) anti-inflammatory activity (inhibition of transcription proinflammatory factors transcription) and may inhibit the deleterious effects of the cytokine storm and tissue damage present in COVID-19. In addition, vitamin D demonstrated a relevant role on glucose-treated monocytes, lowering the risk of oxidative stress and the release of IL-8 and CCL-2 by monocytes. This can be relevant since diabetes is considered a risk factor in COVID-19 patients, in addition to the fact that monocytes glycolysis is a mechanism used by SARS-CoV-2 to promote inhibition of T cells and tissue damage in the lungs.

Taking into account the anti-inflammatory and immunoprotective role that nutrients play in the pulmonary mucosa already discussed in the course of this review, it is not absurd to think that nutrients can be used as an important strategy against SARS-CoV-2 infection (Figure 3). Indeed, some studies, mostly using vitamins and antioxidant nutrients or demonstrating their deficiencies, have already shown some effect during COVID-19, as we describe below.

Perhaps the most prominent vitamin in this context is vitamin D. Many studies have already identified that there is a high incidence of vitamin D deficiency in patients with SARS-CoV-2 infection (220–223). In addition, these studies have also shown a relationship between the level of vitamin D deficiency and the severity of COVID-19. There have been many hypotheses suggested for this relationship, since the anti-inflammatory capacity of vitamin D (223) is very important in a pathology characterized by a proinflammatory cytokine storm (224) that worsens the patient's clinical condition until the possibility of affecting the need for oxygen-support therapy in patients with COVID-19 (222). This deficiency is also related to the risk of mortality from the disease (221). In an *in vitro* study with high glucose-treated monocytes, combined supplementation with vitamin D and l-cysteine was effective in lowering the risk of oxidative stress and the release of IL-8 and C–C motif chemokine ligand 2 (CCL-2) by monocytes (225). This can be relevant in patients with type 2 diabetes and COVID-19 infection, since diabetes is considered a risk factor (226). Moreover, it was recently demonstrated that monocytes play an important role in COVID-19 pathogenicity, since SARS-CoV-2 infection triggers mitochondrial ROS production in monocytes and promotes glycolysis, inhibiting the T cell response and epithelial-cell survival in the lungs (227). Thus, the ability of vitamin D to suppress glucose-treated monocytes can be very important during SARS-CoV-2 infection.

Vitamins A, C and E have also demonstrated some importance in the prognosis of patients with COVID-19, since their deficiencies have been reported, especially in the most severe COVID-19 patients (228). Possibly, the antiviral and anti-inflammatory activity exerted by these nutrients must be impaired in those patients whose vitamin deficiency is more pronounced. In this way, vitamin supplementation in these patients is strongly suggested (229).

Another kind of nutrient deficiency in COVID-19 patients is minerals with antioxidant activity, such as selenium, zinc, magnesium, and copper (228, 230) which are essential in controlling the oxidative stress induced by SARS-CoV-2 infection.

Among flavonoids, naringenin has been shown to be a powerful inhibitor of SARS-CoV-2 infection *in vitro* (231) decreasing viral replication in Vero E6 lineage cells, demonstrating an important role in the control of viral load. In this way, naringenin can be a possible component in the treatment of patients with SARS-CoV-2 infection.

All of these data show that nutrients, in general, play an important role in the control of SARS-CoV-2 infection, which can be used as treatment strategies that may reduce the length of hospital stays and the need for respiratory support in these patients. However, more studies need to be carried out to better define the role that each nutrient may have in COVID-19 prognosis, given the vast anti-inflammatory and antiviral action that each nutrient can exert on the lung environment and the immune system in general.

## Conclusion

The nutrients addressed in this review, in addition to their nutritional role, have a relevant role in maintaining lung health; therefore, adequate consumption of these nutrients is essential to promote an efficient immune response in the control of inflammatory diseases and infections. Moreover, the *in vitro* use of some nutrients with antiviral activity has been shown to be efficient against SARS-CoV-2 infections, which highlights the importance of these components in the current moment of the pandemic that we are facing.

## Author Contributions

SG-S and LO performed conception, and write and review. FT performed conception, write, and illustration and review. MS performed conception and review. AD performed review. All authors contributed to the article and approved the submitted version.

## Funding

This work was supported by the Laboratório de Investigação Médica, Unidade 56, Department of Dermatology, School of Medicine, University of São Paulo, Brazil; Fundação de Amparo à Pesquisa do Estado de São Paulo (FAPESP/nos. 2018/18230-6 and 2019/22448-0) and Coordenação de Aperfeiçoamento de Pessoal de Nível Superior—CAPES: 88887.503842/2020-00–AUXPE no. 689/2020.

## Conflict of Interest

The authors declare that the research was conducted in the absence of any commercial or financial relationships that could be construed as a potential conflict of interest.

## Publisher's Note

All claims expressed in this article are solely those of the authors and do not necessarily represent those of their affiliated organizations, or those of the publisher, the editors and the reviewers. Any product that may be evaluated in this article, or claim that may be made by its manufacturer, is not guaranteed or endorsed by the publisher.

## Abbreviations

AP-1: activator protein 1
ATP: adenosine triphosphate
BALT: bronchial-associated lymphoid tissue
BCR: B-cell receptor
Ca^2+^: calcium
CCL-2: C–C motif chemokine ligand 2
CCR9: C–C motif chemokine receptor 9
CD: cluster of differentiation
COPD: chronic obstructive pulmonary disease
COVID-19: coronavirus disease 2019
CRP: C-reactive protein
DAMPs: damage-associated molecular patterns
DCs: dendritic cells
DNA: deoxyribonucleic acid
EC: epicatechin
ECG: epicatechin-3-gallate
EGC: epigallocatechin
EGCG: epigallocatechin-3-gallate
FcαR: Fc alpha receptor
FOXP3: forkhead box transcription factor P3
GATA3: GATA-binding protein 3
GSH-Px: glutathione peroxidase
HATs: histone acetyltransferases
HCMV: *human cytomegalovirus*
HDAC: histone deacetylase
hTBE: tracheobronchial epithelium
IFN: interferon
IFNAR: interferon alpha and beta receptor
Ig: immunoglobulin
IKK: IκB kinase
IL: interleukin
ILCs: innate lymphoid cells
ISGs: interferon-stimulated genes
IκB: nuclear factor of kappa light polypeptide gene enhancer in B-cells inhibitor
JAK/STAT: janus kinase / signal transducer and activator of transcription
MAMPs: microbial-associated molecular patterns
MgSO^4^: magnesium sulfate
miRNAs: microRNA
NEBs: neuroepithelial bodies
NF-κB: nuclear factor kappa-light-chain-enhancer of activated B cells
NK: natural killer cells
NLR: nucleotide-binding oligomerization domain OR Nod-like receptor
Nrf2: nuclear-related factor 2
PAMPs: pathogen-associated molecular patterns
PBMC: peripheral blood mononuclear cell
PGC: peroxisome proliferator-activated receptor-gamma coactivator
PGE 2: prostaglandin E 2
PNECs: pulmonary neuroendocrine cells
PRRs: pattern recognition receptors
RA: retinoic acid
RDS: respiratory distress syndrome
RIG-1: retinoic acid-inducible gene I
RNA: ribonucleic acid
RORγt: retinoic acid-related orphan receptor gamma t
ROS: oxygen species
RSV: respiratory syncytial virus
SARS-CoV-2: severe acute respiratory syndrome coronavirus 2
SCFAs: short-chain fatty acids
SFB: segmented filamentous bacteria
SIRPα: signal regulatory protein α
SIRT1: sirtuin 1
TGF-β: transforming growth factor β
Th: T helper
TLRs: Toll-like receptors
TNFR: tumor necrosis factor receptor
Tregs: regulatory T cells
TREM2: triggering receptor expressed on myeloid cells 2
WHO: World Health Organization
ZIP10: zinc transporter 10
ω: omega.
